# Mesenchymal stromal cells and neuroinflammation: a multimodal approach to neuroprotection and future therapeutic horizons

**DOI:** 10.1186/s40035-026-00554-4

**Published:** 2026-05-18

**Authors:** Alexandro Angelo Bufi, Andrea Papait, Peter Ponsaerts, Antonietta Rosa Silini, Ornella Parolini

**Affiliations:** 1 Department of Life Science and Public Health, Università Cattolica del Sacro Cuore, 00168 Rome, Italy; 2 Laboratory of Experimental Hematology, Vaccine and Infectious Disease Institute (Vaxinfectio), University of Antwerp, 2610 Wilrijk, Belgium; 3 Fondazione Policlinico Universitario Agostino Gemelli, IRCCS, 00168 Rome, Italy; 4 Centro Di Ricerca E. Menni, Fondazione Poliambulanza Istituto Ospedaliero, 25124 Brescia, Italy; 5 Fondazione IRCCS Casa Sollievo Della Sofferenza, San Giovanni Rotondo, 71013 Foggia, Italy

**Keywords:** Neuroinflammation, Neurodegeneration, Mesenchymal stromal cells, Immunomodulation, Regenerative medicine, Stem cells

## Abstract

As the global population ages, neurodegenerative and neuroinflammatory diseases are becoming a rapidly growing public health challenge, with available interventions remaining largely symptomatic and often only modestly affecting long-term disease progression. Therapies involving mesenchymal stromal cells (MSCs) have attracted substantial attention as a potential clinical therapeutic strategy across chronic central nervous system (CNS) disorders, due to their multifaceted ability to modulate immune response and confer neuroprotection. While initially explored for their multilineage differentiation potential, MSCs are now predominantly recognized for their paracrine functions, including secretion of soluble factors and extracellular vesicles. These acellular mediators induce diverse neuroprotective effects by attenuating neuroinflammation, stabilizing the blood–brain barrier, reprogramming glial and lymphocyte activity, and delivering regulatory microRNAs that modulate neuronal apoptosis and inflammatory gene networks. In this review, we summarize molecular evidence from in vitro and in vivo preclinical models, and early clinical investigations that demonstrate how tissue source and immunobiological plasticity shape the efficacy of MSCs. We further highlight emerging trends toward acellular MSC-derived therapies, offering a mechanistically versatile platform for therapeutic interventions for common neurodegenerative and neuroinflammatory disorders of the CNS, particularly Alzheimer’s disease, Parkinson’s disease and multiple sclerosis, a primary autoimmune demyelinating disorder.

**Graphical abstract:**

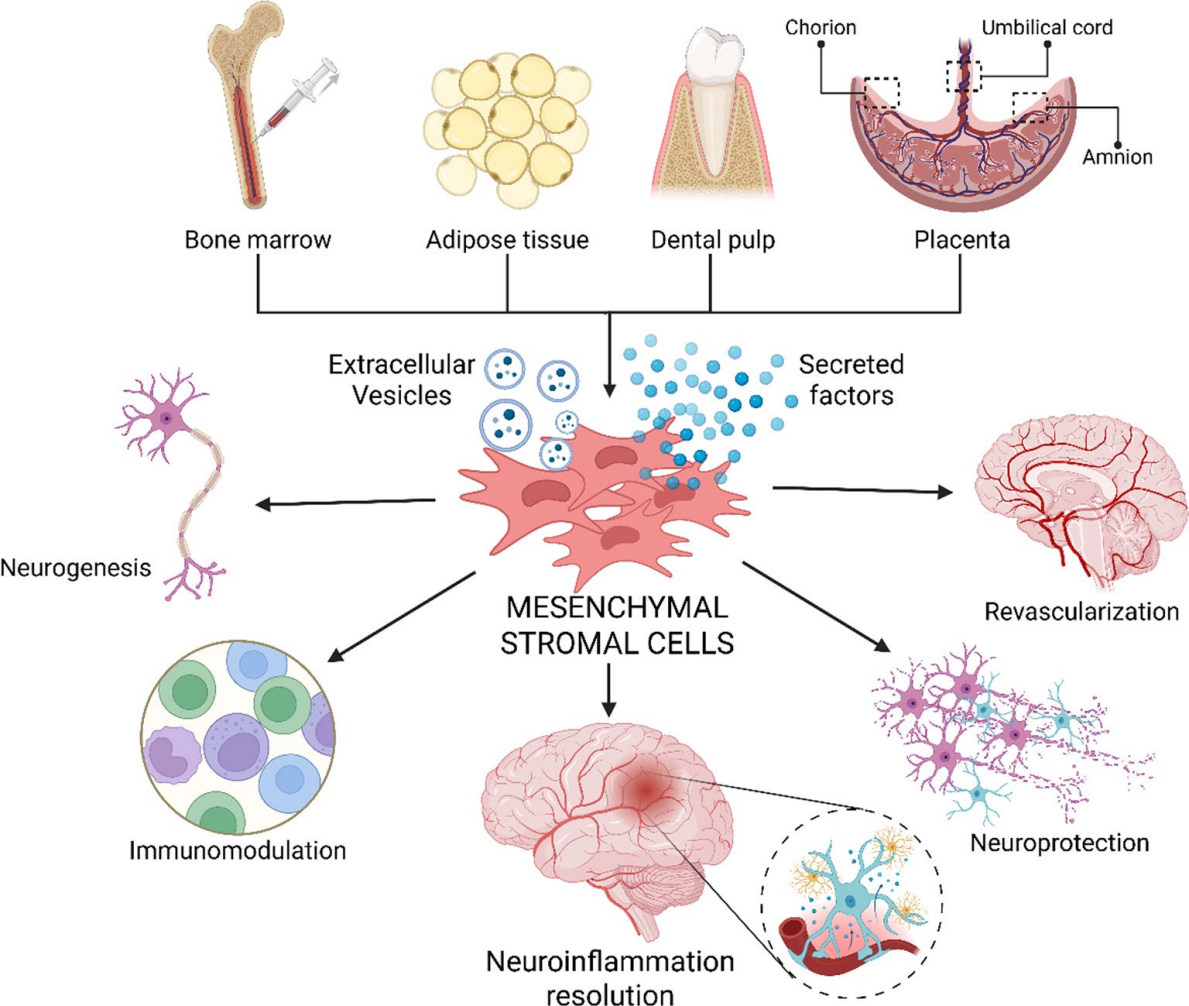

## Introduction

Advances in medicine have markedly increased life expectancy [1], but this demographic shift has simultaneously contributed to a rise in age-associated neurodegenerative diseases (NNDs), now a major global health burden [2]. NNDs comprise heterogeneous disorders characterized by progressive and region-specific neuronal degeneration within the central nervous system (CNS) [3]. Despite distinct etiologies and disease-specific pathological signatures, these conditions usually share some of the following convergent mechanisms: synaptic dysfunction, aberrant proteostasis with protein aggregation, cytoskeletal disruption, metabolic imbalance, genomic damage, and chronic neuroinflammation [4], the latter of which is now recognized as a central pathological feature [3]. Accordingly, elucidating the cellular and molecular underpinnings of neuroinflammatory interactions is a critical step toward development of new therapeutic strategies [5]. Mesenchymal stromal cells (MSCs) are among the most compelling strategies, and their therapeutic actions are now known to be largely mediated by their paracrine signalling [6, 7]. Acellular derivatives, such as the full repertoire of factors secreted by MSCs (secretome), or the isolated extracellular vesicles (EVs) [8–10], contain immunomodulatory, neurotrophic, and anti-apoptotic factors, enabling a multitarget intervention capable of reprogramming the neuroinflammatory milieu [11–13]. This review will dissect the dynamic interplay between MSC-derived products and the neurodegenerative and inflammatory environment, with an emphasis on the mechanistic basis for their pleiotropic therapeutic effects.

## Neuroinflammation in CNS disorders

Neuroinflammation comprises the activation of microglia and astrocytes, increased secretion of pro-inflammatory cytokines, and infiltration of peripheral immune cells into the CNS, ultimately leading to localized tissue damage [14]. Under physiological conditions, resident glial cells support CNS homeostasis by sensing perturbations, clearing cellular debris, and contributing to host defense. In contrast, persistent or dysregulated activation of these cells can drive chronic neuroinflammation, a common feature of multiple CNS disorders [3, 15]. In response to inflammatory stress, neurons can also adopt a “neuroinflammatory” phenotype, characterized by oxidative stress, increased susceptibility to apoptosis, and release of danger-associated mediators that further recruit and activate glia [3, 16]. Overall, microglia, astrocytes, and infiltrating immune cells are the primary mediators, releasing cytokines and reactive oxygen species (ROS) [17], which in turn amplify neuronal stress responses and inflammation-associated neuronal dysfunction.

## Glial and endothelial dynamics in the inflammatory CNS microenvironment

### Microglia surveillance and activation states

Microglia can detect pathogen- and damage-associated molecular patterns through the expression of diverse pattern recognition receptors, especially Toll-like receptors [18, 19]. They can phagocytose cell debris, infectious agents, and apoptotic bodies, thereby maintaining CNS integrity [20]. Beyond immune surveillance, microglia contribute to tissue homeostasis, synaptic remodeling, and myelination [21, 22]. Rather than fitting into rigid M1/M2 categories, microglia display a spectrum of activation states [23]; for clarity, we refer here to “M1‑like” and “M2‑like”. The M1‑like microglia, triggered by interferon‑gamma or TLR engagement [24], produce interleukin (IL)-1β, tumor necrosis factor alpha (TNF‑α), IL‑6, nitric oxide (NO), and metalloproteinases (MMPs) [18, 25]. Conversely, the M2-like states promote resolution and repair via IL-10 and transforming growth factor-beta (TGF-β) [26]. Under physiological conditions, microglial activation is transient and resolves once the triggering stimulus is removed [27]. However, in chronic NNDs, persistent stimuli maintain microglia in a sustained pro-inflammatory and neurotoxic phenotype [28]. In Alzheimer’s disease (AD), for example, β-amyloid (Aβ) plaques and hyperphosphorylated tau induce microglial activation, with resulting tissue damage releasing damage-associated molecular patterns that further amplify inflammation [29, 30]. Microglial activation can even promote Aβ production and aggregation through upregulation of IFITM3 (interferon-induced transmembrane protein 3) [31], or by iron release [32, 33]. Microglial activation can also drive tau pathology, as microglia-derived exosomes transport hyperphosphorylated tau, promote prion-like propagation of neurofibrillary tangles and exacerbate neurodegeneration [34, 35]. Similarly, in Parkinson’s disease (PD), α‑synuclein, a presynaptic protein with physiological roles in synaptic and mitochondrial functions, forms pathogenic complexes upon misfolding and aggregation [36]. In PD models, aggregated α-synuclein robustly engages microglia/monocytes, promoting an inflammatory response characterized by increased secretion of pro-inflammatory mediators. Notably, the α-synuclein-induced neurotoxicity is markedly reduced when microglia are depleted or absent, indicating that microglial activation is crucial for neuronal injury [37–39].

### Astrocyte dynamics: from support to reactivity

Astrocytes are now recognized as active participants in neuroinflammation, responding to CNS damage and stress signals [40]. While they normally support CNS homeostasis by maintaining the blood–brain barrier (BBB) integrity, modulating synaptic plasticity, regulating ion and fluid balance, clearing apoptotic bodies and pathogens, and forming glial scars [41–43], inflammatory cues can convert them into neurotoxic reactive astrocytes [44]. They can release IL-1β, TNF-α, NO, and the complement component 3 (C3) [45, 46], and have increased expression of glial fibrillary acidic protein (GFAP), IL-17 receptor, and tropomyosin receptor kinase B [47]. Conversely, resolution-promoting astrocytes support tissue repair and neurogenesis, and ameliorate inflammation [48] by producing anti-inflammatory cytokines such as IL-4, IL-10, TGF-β, and neurotrophic factors including brain derived neurotrophic factor (BDNF) and glial-cell derived neurotrophic factor (GDNF) [46, 49]. Signal transducer and activator of transcription 3 (STAT3), an activated downstream of BDNF signaling, is a key regulator of astrocyte differentiation. Loss of STAT3 function leads to increased immune cell infiltration, neuronal loss, and demyelination [50]. In spinal cord injury models, STAT3 deficiency impedes glial scar formation, permitting uncontrolled tissue damage [51]. Astrocytes and microglia engage in bidirectional communications. Astrocyte-derived IL-1β, complement proteins, NO, chondroitin sulfate proteoglycans (CSPGs), and chemokines like C–C motif chemokine ligand 2 (CCL2) and C-X-C motif chemokine ligand 10, can potentiate microglial neurotoxic activation [52]. Conversely, microglia release TNF-α, IL-1α, and complement component 1q, which sustain astrocyte activation [52]. In AD models, the expression of astrocytic marker GFAP is increased, indicating the emergence of disease-associated astrocytes (DAAs). DAAs exhibit altered expression of genes implicated in inflammation and amyloid metabolism [53, 54], paralleled by reduced expression of genes critical for neuronal support [53]. In PD models, especially the MPTP (1-methyl-4-phenyl-1,2,3,6-tetrahydropyridine) model, a significant increase of C3^+^ reactive astrocytes is observed near regions of dopaminergic neuron loss [55]. Chronic administration of BSSG (β-sitosterol β-D-glucoside) also increases the population of astrocytes co-expressing C3 with GFAP or S100B [56, 57].

### BBB integrity in health and disease

**Fig. 1:**
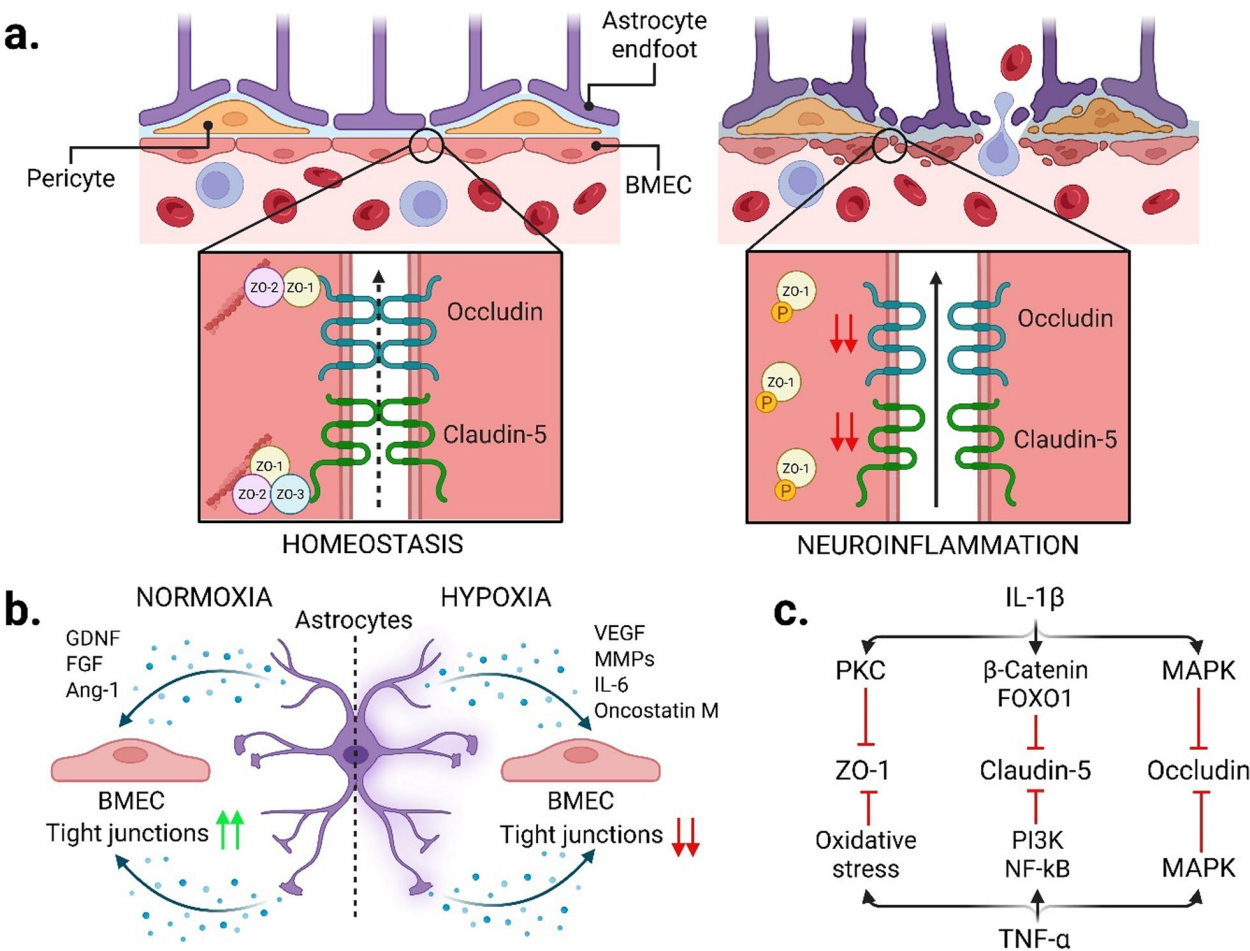
Mechanisms of BBB disruption under neuroinflammatory conditions. **a** In homeostatic conditions, BBB integrity is preserved by tightly regulated interactions between BMECs, pericytes, and astrocytic endfeet, supported by tight junction proteins such as occludin, claudin-5, and ZO-1/2/3. Neuroinflammatory stimuli compromise tight junction architecture, increasing paracellular permeability. **b** Astrocytes modulate BMEC phenotype in response to oxygen availability. Under normoxia, astrocyte-derived GDNF, FGF, and Ang-1 enhance tight junction expression. Conversely, hypoxic or inflammatory conditions stimulate astrocytes to release VEGF, MMPs, IL-6, and oncostatin M, which weaken tight junction integrity and promote BBB breakdown. **c** Proinflammatory cytokines such as IL-1β and TNF-α disrupt tight junction expression via multiple intracellular signalling pathways, including PKC, MAPK, and PI3K/NF-κB. These cascades lead to oxidative stress and transcriptional repression of ZO-1, occludin, and claudin-5, contributing to BBB dysfunction. Image created with Biorender.com

The BBB, consisting of endothelial cells, pericytes, and astrocytic endfeet, forms a semi-permeable barrier that tightly regulates CNS exchange with the circulation [58]. The integrity of the BBB is mediated by tight junctions connecting brain microvascular endothelial cells (BMECs) [59, 60]. During neuroinflammation, BBB integrity is compromised **(**Fig. 1a**)**. Pro-inflammatory glial activation negatively regulates expression of tight-junction proteins, promoting barrier leakage and allowing circulating immune cells and soluble inflammatory mediators to enter the CNS, thereby amplifying glial reaction and tissue injury in a self-sustained, harmful loop [61]. Under physiological conditions, astrocytes reinforce BBB impermeability by releasing GDNF, fibroblast growth factor (FGF), and angiopoietin-1 (Ang-1) [62–64]. However, under oxygen and glucose deprivation, they upregulate MMPs, particularly MMP9, and vascular endothelial growth factor (VEGF), which degrade tight-junctions [65, 66]. Cytokines such as IL-6 and oncostatin M elevated in multiple sclerosis (MS), disrupt the barrier by reducing claudin-5 expression [67–69] **(**Fig. 1b**)**. Microglial activation also increases BBB permeability. Lipopolysaccharide-stimulated microglia increase the permeability of the barrier [70], and this effect can be reversed by reducing ROS production [70, 71] or blocking TNF-α with a neutralizing antibody [72]. Exposure of BMECs to IL-1β induces the phosphorylation of zonula occludens 1 (ZO-1) through activation of protein kinase C (PKC), which leads to its relocalization from the cell membrane to the cytoplasm*,* disrupting its interactions with other tight-junction proteins [73, 74]. Similarly, prolonged exposure to IL-1β downregulates the expression of claudin-5 by promoting the nuclear translocation of β-catenin and FOXO1 (forkhead box protein O1) [75]. It also reduces occludin expression via the mitogen-activated protein kinase (MAPK) signaling [76]. TNF-α represses claudin-5 promoter activity through nuclear factor kappa-light-chain enhancer of activated B cells (NF-κB) signaling and phosphoinositide 3-kinase (PI3K) pathway [77, 78]. TNF-α also lowers occludin [76] and ZO-1 [79, 80] expression **(**Fig. 1c**)**, while indirectly inducing BMEC release of IL-6 [81] and SPARC (the secreted protein acidic and rich in cysteine), an extracellular matrix remodeler that hinders the correct function of tight junctions [82].

## Peripheral immunity involvement in CNS tissue damage

Neuroinflammation is not solely driven by CNS resident cells. Peripheral immune cells also play essential roles in initiating and amplifying the neuroinflammatory processes [83, 84]. Although dissecting the exact contribution of each cell across NNDs is complex, MS may serve as a paradigmatic example due to its autoinflammatory nature driven by autoreactive CD4^+^ T lymphocytes targeting CNS antigens [85]. Unlike PD or AD, where neuroinflammation is generally considered secondary and contributory to neurodegeneration, MS is a primary chronic autoimmune and demyelinating disorder of the CNS. Although the exact trigger for the autoinflammatory cascade remains incompletely understood, current evidence suggests a complex interplay of genetic susceptibility and environmental factors that promotes the activation of peripheral autoreactive lymphocytes, leading to loss of immune tolerance and subsequent breach of BBB [86]. In experimental autoimmune encephalitis (EAE), peripheral immunization with myelin basic protein (MBP) triggers a CD4^+^ T cell–mediated immune response: activated cells migrate to the draining lymph nodes [87], differentiate into Th1 and Th17 subsets [88], and secrete pro-inflammatory cytokines, such as IFN-γ, IL-2 and TNF-α, which show increased cerebrospinal fluid (CSF) levels correlating with disease severity [89]. Recruitment of autoreactive T cells is facilitated by the upregulation of integrins, LFA-1 (lymphocyte function-associated antigen 1) and VLA-4 (very late antigen-4), that enable adhesion and transmigration across the BBB [28]. EAE can be induced by adoptive transfer of CD4⁺ T cells specific to CNS antigens, or by using transgenic mice expressing T cell receptors (TCRs) and human HLA class II molecules recognizing myelin-derived epitopes [90]. Th17 cells exert particularly potent neurotoxic effects and induce more severe EAE upon adoptive transfer compared to Th1 cells [91]. They produce IL-17 and IL-22 to amplify inflammation via production of granulocyte–macrophage colony-stimulating factor, IL-6, and IL-8 [92], and can directly cause injury to neuronal axons through TCR-independent contact that disrupts intracellular calcium homeostasis [93].

CD8^+^ T cells specific to CNS antigens are abundant at demyelination sites [94, 95] and become activated through interactions with antigen presenting cells (APCs) [96]. Since neuronal axons constitutively express MHC-I, they are particularly vulnerable to CD8⁺ T cell-mediated cytotoxicity, a process exacerbated by the fact that CD8^+^ T cells often outnumber CD4^+^ T cells within MS lesions [97]. The inflammatory environment in MS further upregulates MHC-I on neurons and oligodendrocytes, enhancing their susceptibility to immune attack [98, 99]. In addition to releasing pro-inflammatory cytokines [100], CD8^+^ T cells release perforin and granzymes A/B, which induce direct cytolytic damage. Finally, they can trigger caspase-dependent apoptosis via FAS signaling on neuronal membranes and axons [101]. Despite this cytotoxicity, CD8^+^ T cells can also exert regulatory functions. Their depletion worsens EAE in CD28^−^/^−^ mice, while adoptive transfer of CD8^+^CD28^−^ T cells attenuates MS [102]. Moreover, MOG (myelin oligodendrocyte glycoprotein)-stimulated CD8^+^ T cells can suppress EAE by selectively eliminating pathogenic CD4^+^ T cells [103].

CD4^+^ Tregs antagonize Th1, Th17, and cytotoxic CD8^+^ T lymphocytes. In MS, mutations in CD25 and cytotoxic T-lymphocytes antigen 4 (CTLA-4), along with reduced expression of forkhead box P3 (FoxP3) and IL-10, impair Treg functions [104]. Restoring Treg activity, either by transferring FoxP3^+^ Tregs into EAE mice [105], or stimulating the function of CD28^+^ Tregs, attenuates the disease course, suggesting the correlation between decreased Treg numbers and MS symptoms [106].

**Fig. 2:**
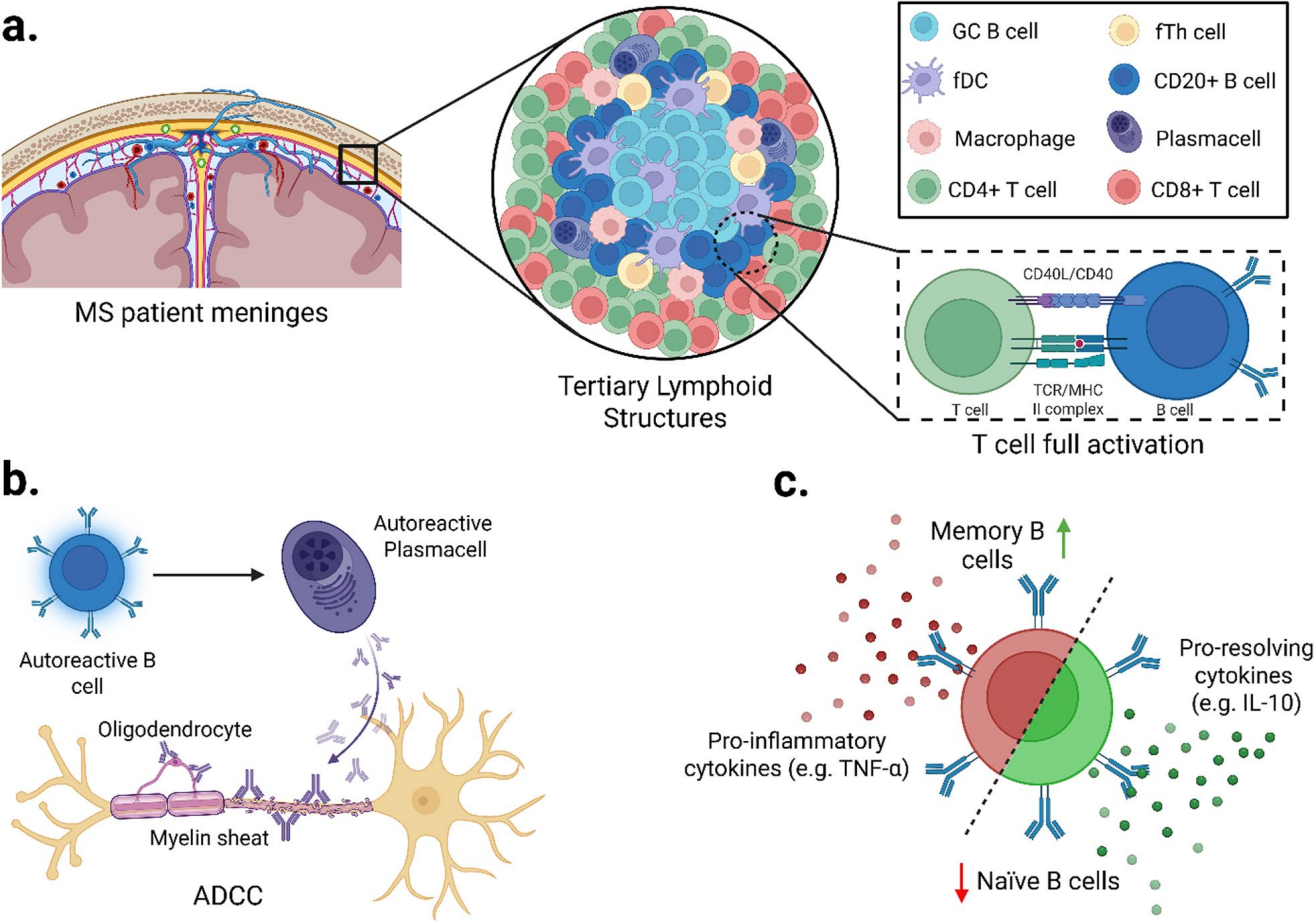
Pathogenic roles of B cells in MS development. **a** In the meninges of MS patients, ectopic tertiary lymphoid structures form and support local immune activation. T cell–B cell interactions via TCR–MHC-II complexes and co-stimulatory molecules, such as CD40. They also promote full T cell activation and local antigen presentation. **b** Autoreactive B cells differentiate into plasma cells that produce pathogenic antibodies targeting oligodendrocytes and myelin sheath, therefore contributing to NK and T cell-driven ADCC, which leads to neuron demyelination. **c** Imbalance between pro-inflammatory and regulatory B cell responses contributes to disease progression. While memory B cells secrete TNF-α and other pro-inflammatory cytokines, regulatory and naïve B cells release IL-10 and promote immune resolution. A skewed ratio favouring memory over naïve or regulatory B cells characterizes active MS lesions

B cells also contribute to the MS pathogenesis, as shown by the accumulation of B lymphocytes, plasma cells, and immunoglobulins within brain lesions and in the CSF [107]. B cells enhance T cell activation by presenting antigens and expressing co-stimulatory factors, including CD40, CD80, and CD86 [108, 109]. They also form tertiary lymphoid structures within the meninges of MS patients, enabling local T cell activation and expansion [110] **(**Fig. 2a**)**. Autoreactive B cells exacerbate tissue degeneration by differentiating into plasma cells that produce antibodies targeting myelin and oligodendrocytes, promoting antibody-dependent cell-mediated cytotoxicity (ADCC) by natural killer (NK) and cytotoxic T cells [111] **(**Fig. 2b**)**. Furthermore, MS switches the cytokine profiles of human B cells from the native pool (IL-10 production by naïve B cells) to the memory pool (secretion of TNF-α by memory B cells), reducing the regulatory capacity [112] **(**Fig. 2c**)**.

At the peripheral level, monocytes from MS patients display a pro-inflammatory profile, with elevated basal production of IL-1β, TNF-α, IL-6 and IL-8 [113]. Classical CD14^+^ CD16^−^ monocytes expressing C–C chemokine receptor 2 (CCR2) invade the CNS along a CCL2 gradient, and genetic ablation of CCR2 in EAE models prevents lymphocyte infiltration, confirming its critical role in early disease pathogenesis [114]. Monocyte entry into the meninges is one of the earliest immunological events in EAE, intensifying until clinical symptoms manifest [115]. Beyond infiltration, monocytes and myeloid cells act as APCs that sustain neuroinflammation by presenting CNS autoantigens [116]. However, mixed outcomes from CCR2-targeting clinical trials suggest that monocytes are not exclusively pathogenic [117], but may also support tissue repair by replenishing macrophage populations [118, 119]. In neurodegeneration, extracellular iron accumulation, caused by myelin degradation and oligodendrocyte death and associated with worse cognitive decline [120, 121], is mitigated by microglia and macrophages, which uniquely express transferrin receptor, enabling them to scavenge iron and limit ROS production [122].

The role of NK cells in MS pathogenesis remains incompletely defined, as they may exert both cytotoxic and protective effects. While classically involved in eliminating virally infected, tumor, or stressed cells, NK cells can also limit neuroinflammation by killing antigen-presenting dendritic cells [123] and autoreactive T lymphocytes [124, 125]. Moreover, NK cells can restrict the differentiation of naïve T cells into Th1 and Th17 clones targeting myelin antigens [126]. This protective activity depends in part on CD226 (DNAX accessory molecule-1, DNAM-1) [127, 128]. An MS-associated risk allele reduces CD226 expression and impairs NK-mediated clearance of autoreactive T cells [129, 130]. CD56^bright^ NK cells, which are less mature, highly IFN-γ-producing [131], and enriched for granzyme K, accumulate at T-cell infiltration sites in MS lesions [132]. MS skews NK maturation toward the CD56^bright^ subset, which is the major NK cell subset compared to CD56^dim^ NK cells in the CSF [132, 133]. Also, activated T cells upregulate ligands for the natural killer group 2D (NKG2D) receptor. This makes them susceptible to NK-mediated cytotoxicity [134], a process in which granzyme K plays a major role [135]. NK cells also target microglia through NKG2D and the natural cytotoxicity receptor NKp46 [136], as well as immature myeloid-derived cells [137, 138]. Interestingly, several MS therapies modulate NK cell cytotoxicity, enhancing their ability to eliminate antigen-presenting dendritic cells [123].

## MSCs in CNS repair: mechanisms of action and therapeutic promise

MSCs have attracted significant attention for their capacity to modulate the CNS microenvironment and promote functional recovery following injury [139–141]. The International Society for Cell & Gene Therapy (ISCT) defined MSCs as plastic-adherent, multipotent cells that express CD105, CD73, and CD90, but lack hematopoietic and immune markers (CD45, CD34, HLA-DR) [142]. MSCs also exhibit trilineage differentiation potential, so they can be differentiated in vitro into bone, adipose, and cartilage cells [143]. They can be isolated from a wide range of tissues. Bone marrow-derived MSCs (BM-MSCs) were the first to be identified, and therefore are the most extensively characterized, and remain the prototypical source [144]. Their major limitation lies in their localization, as bone marrow aspiration is inherently invasive and limits routine sample collection. In contrast, adipose-derived MSCs (AD-MSCs) [145] and peripheral blood-derived MSCs offer advantages in terms of accessibility, reduced procedural risk, and autologous application [146]. Over time, the spectrum of harvestable tissues has expanded, with neural crest-derived MSC populations identified in oral mucosa [147] and dental pulp [148]. Particularly advantageous are perinatal tissues, which have rapidly emerged as a highly convenient source of MSCs [149]. As these tissues are normally discarded after birth, their use circumvents the need for additional invasive procedures, providing an ethically favorable and readily available reservoir for MSC isolation [149]. Perinatal MSCs exhibit high proliferative capacity and potent immunomodulatory activity, and produce neurotrophic factors such as BDNF, GDNF, and VEGF. These cells have shown benefit in models of ischemic stroke and Huntington’s disease [150, 151].

Attempting to choose a single source of MSCs as universally superior for the treatment of human diseases is considered outdated. It has become evident that each MSC population displays distinct advantages and limitations that manifest differently depending on the pathological context [152]. From a clinical standpoint, MSCs have demonstrated an exceptional safety profile [153, 154]. In several phase I clinical trials aimed at the treatment of NNDs, MSC infusion was associated with very limited graft-related reactions and a remarkably low incidence of adverse events [155–158]. A more in-depth discussion about clinical trials in NNDs is found in a separate paragraph.

## Principal axis of MSC-mediated immunomodulation

The promising safety profile shown by MSCs relies on several molecular mechanisms, with the very low immunogenicity being the most prominent [159, 160]. This intrinsic feature prompted the hypothesis that their clinical utility could rely on their ability to modulate dysregulated immune responses [8, 11]. Current consensus emphasizes that MSC-mediated neurorepair arises predominantly from paracrine mechanisms rather than neuronal replacement [6]. Strikingly, this regulatory function does not depend on cellular viability; it persists even when MSCs are metabolically inactivated, fragmented, or apoptotic, underscoring the paracrine nature of their bioactivity [13, 161]. Apoptotic MSCs generated ex vivo retain their immunoregulatory capacity. After infusion, they are phagocytosed by host macrophages, which subsequently upregulate indoleamine 2,3-dioxygenase (IDO), a rate-limiting enzyme in tryptophan metabolism critical for immunosuppression [162]. These observations have contributed to the development of cell-free therapeutics that aim to preserve the immunomodulatory and trophic effects of MSCs while improving the standardization, scalability, and safety [163, 164].

MSCs exert multifaceted effects on innate immune cells. Through secretion of soluble mediators, such as Prostaglandin E₂ (PGE_2_) [165, 166], IDO [165, 167], hepatocyte growth factor (HGF) [168], TGF-β [169], and interleukin-1 receptor antagonist (IL-1RA) [170], MSCs can reprogram microglia and monocytes toward the neuroprotective M2-like phenotype, characterized by enhanced secretion of IL-10 and arginase-1, and downregulation of pro-inflammatory effectors [171, 172]. This reprogramming also occurs at the metabolic level and is further supported by exosomal delivery of microRNAs that modulate gene expression networks [173]. The M2 monocytes also downregulate co-stimulatory molecules, reducing pro-inflammatory T lymphocytes [174] and promoting the differentiation of Tregs, extending immunoregulatory effects to other immune cells [174, 175]. Monocytes co-cultured with umbilical cord-derived MSCs (UC-MSCs) show reduced expression of HLA-DR/DP/DQ and CD86, as well as impaired antigen presentation capacity and phagocytosis [176]. UC-MSCs also reprogram monocytes to acquire the CD14^+^ CD16^+^CD206^+^ phenotype, characterized by IL-10 secretion and upregulation of programmed death-ligand 1 [177].

MSCs can also modulate adaptive immune responses. MSCs suppress T-cell proliferation through both contact-dependent and -independent mechanisms. Fas ligand (FasL)-mediated apoptosis has been shown in co-culture models [178], while exosomes can arrest T-cell cycling by modulating expression of cyclin-dependent kinase 2 and cyclin-dependent kinase inhibitor 1B [179]. Interestingly, MSCs may also promote T-cell quiescence by downregulating Fas expression, maintaining cells in the G₀ phase without inducing apoptosis [180]. Furthermore, MSCs modulate T-cell polarization by inhibiting Th1 and Th17 differentiation while promoting Th2 and regulatory T-cell expansion, a shift mediated by factors such as IDO and HGF [181, 182]. By transferring their mitochondria via actin-based tunnelling nanotubes, BM-MSCs can convert Th17 cells into an immunosuppressive Treg phenotype, characterized by the expression of FoxP3, CD25, CTLA-4, and TGF-β1 [183]. UC-MSCs can directly inhibit the activity of Th1 and CD8^+^ T cells by reducing TNF-α and IFN-γ [184], while simultaneously promoting IL-10 secretion [185]. Regarding cytotoxic T cells, our group demonstrated that hAMSCs impede the effector differentiation of naïve CD8⁺ T cells by attenuating phosphorylation of mTOR (mammalian target of rapamycin) and protein kinase B (Akt) and downregulating IL-12Rβ1/IL-2RA (interleukin-12 receptor β1 and RA), thereby inhibiting the STAT4/5 signaling [186].

The effects of MSCs, or their acellular products, on T cells have raised concerns on potential systemic effects on immunity when administered systemically. Intrathecal administration facilitates localized modulation of neuroinflammation within the CNS, whereas intravenous delivery may elicit systemic consequences [187]. MSCs transiently localize to the pulmonary vasculature and secondary lymphoid organs, where they engage host immune populations [188]. These interactions result in the functional licensing of Tregs, which subsequently home to the CNS to suppress inflammation [189]. The induction of peripheral immune tolerance, rather than broad immunosuppression, distinguishes MSCs from conventional pharmacological agents [190]. Notably, despite their potent immunoregulatory effects, MSC therapies have not been associated with an increased risk of opportunistic infections or malignancy, underscoring a favorable safety profile relative to long-term immunosuppression [190–192].

The immunomodulatory actions of MSCs also target B lymphocytes. MSCs inhibit B-cell proliferation and plasma cell differentiation through paracrine factors that modulate the extracellular signal-regulated kinase 1/2 (ERK 1/2) and p38 signaling pathways [193, 194]. Their secretomes, rich in TGF-β and IL-1RA, also favor a regulatory B cell phenotype under specific cytokine exposure, especially after the priming with IFN-γ [195–197]. Additionally, BM-MSC-derived EVs influence B-cell development by modulating PI3K/Akt activity and disrupting the T follicular helper–B cell interactions in early immune responses [184, 198].

Finally, MSC interactions with NK cells remain controversial. While consistently shown to suppress NK proliferation via G_0_/G_1_ arrest and apoptosis [199, 200], the effects of MSCs on NK cytokines vary among studies. Some studies reported enhanced IFN-γ secretion induced by BM-MSCs [201], whereas others demonstrated UC-MSC-mediated suppression through impaired STAT4/NF-κB signaling and activin A-driven downregulation of T-bet, a master transcriptional regulator of IFN-γ [202]. Functionally, MSCs downregulate activating NK receptors, including NKG2D, NKp46, and CD16, while upregulating some inhibitory killer immunoglobulin-like receptors, such as CD158, thereby attenuating NK cytotoxicity [203, 204].

**Fig. 3:**
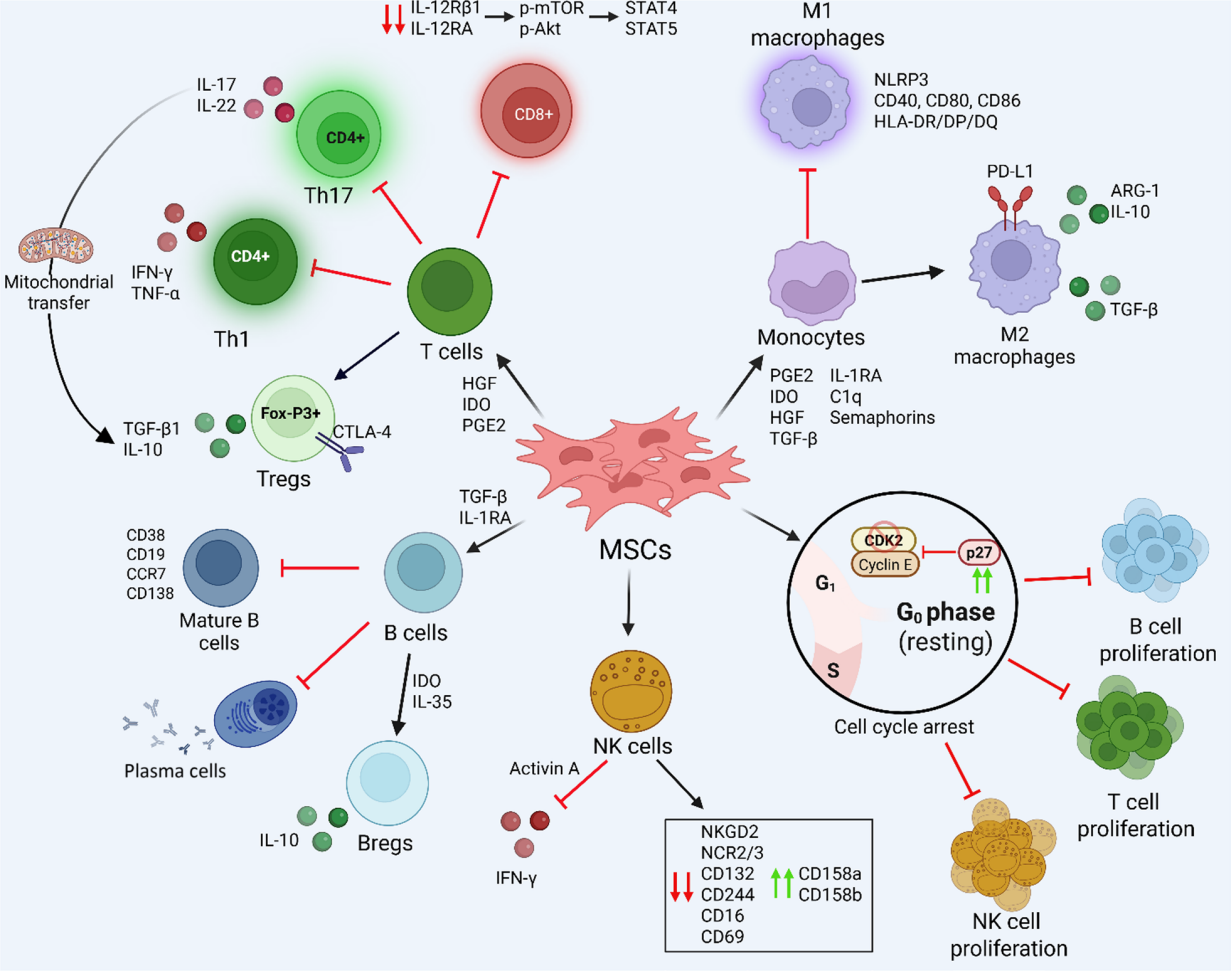
Broad immunomodulatory effects of MSCs on innate and adaptive immunity**.** MSCs exert potent immunoregulatory effects by modulating both phenotype and function of immune cells across the innate and adaptive spectrum. In the T cell compartment, MSCs suppress Th1 and Th17 polarization and reduce CD8⁺ T cell activation and proliferation by releasing immunoregulatory molecules such as IDO, PGE2, and HGF. They concurrently promote the expansion and function of Tregs, supporting the resolution of inflammation through cell–cell contact mechanisms involving CTLA-4 and the secretion of TGF-β and IL-10. MSCs can also impair B cell proliferation, antibody-secreting plasma cell differentiation, and antigen presentation, while fostering the development of IL-10⁺ regulatory B cells via IDO and IL-35. Concerning innate immunity, MSCs inhibit monocyte differentiation into pro-inflammatory M1 macrophages and promote M2 polarization, characterized by PD-L1, TGF-β, and IL-10 expression. MSCs can also interfere with NK cell activation by altering the balance of activating and inhibitory receptor signals and downregulating IFN-γ production, primarily via molecules such as Activin A

Collectively, these findings reveal that the immunomodulatory properties of MSCs can influence the activation and phenotypes of both innate and adaptive immunity. This paradigm supports MSC-based approaches as a potentially useful, safe, and mechanistically versatile strategy in regenerative immunology and neurotherapeutic **(**Fig. 3**)**.

## Neuroprotective and revascularization capacities of MSCs

MSCs exhibit robust neuroprotective properties that enhance neuronal survival and mitigate injury-induced apoptosis. In a neonatal rat model of hypoxic-ischemic encephalopathy, UC-MSCs significantly reduced brain damage by modulating Beclin-2 and caspase-3 signaling pathways, thereby attenuating apoptotic cascades and preserving motor function [205]. Complementary in vitro findings corroborated that neuroprotection was mediated via upregulation of the anti-apoptotic B cell lymphoma 2 (Bcl-2) protein in rat primary neuronal and astrocyte cultures [206]. Similarly, administration of BM-MSCs in stroke-prone spontaneously hypertensive rats led to increased Bcl-2 gene expression, reduced oxidative stress markers such as superoxide anions and lipid peroxidation products, and histological evidence of restoration of hippocampal integrity [207].

Following cerebral ischemia, BBB breakdown precipitates widespread inflammation, neuronal necrosis, and cerebral oedema [208]. Restoration of microvascular perfusion is critical to re-establish oxygen and nutrient supply, thereby supporting neuronal repair [209]. Indeed, clinical evidence has associated higher capillary density with improved post-stroke survival outcomes [210]. MSCs foster revascularization by secreting angiogenic factors such as Ang-1, placental growth factor, and VEGF [211–213]. Mechanistically, MSCs activate Notch signalling in endothelial cells, thereby inducing autocrine VEGF-A production and promoting neovascularization [209]. Furthermore, conditioned medium from BM-MSCs protects human aortic endothelial cells from hypoxia-induced apoptosis and stimulates proliferation via the PI3K pathway [214].

## MSC-derived EVs and their miRNA cargoes

**Fig. 4:**
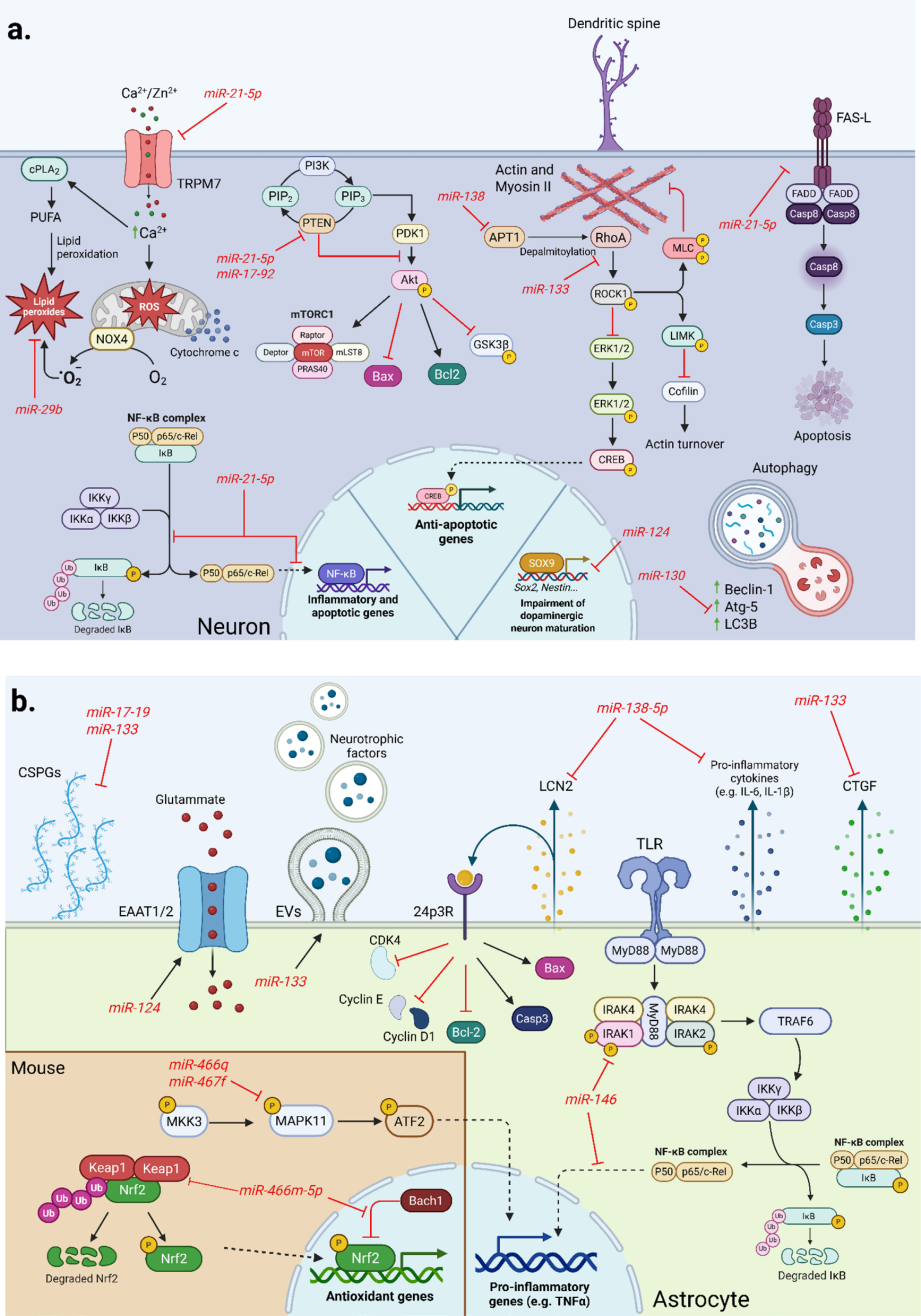
Neuronal and astrocytic pathways modulated by MSC-derived miRNAs. **a** MSC-derived EVs deliver miRNAs that regulate processes involved in survival, inflammation, and synaptic integrity, attenuating mitochondrial stress and apoptosis in injured neurons. miR-21-5p, miR-17–92, and miR-29b inhibit key pro-apoptotic and oxidative stress-related pathways by repressing PTEN, lipid peroxidation, and components of the NF-κB signalling. miR-138 and miR-133 preserve dendritic spine morphology by targeting APT1 and RhoA, leading to the CREB-mediated transcription of anti-apoptotic genes. Mir-124 suppresses SOX9, promoting neuronal maturation, while miR-130 inhibits inflammation, targeting autophagy. **b** EV-delivered miRNAs released by MSCs also regulate astrocytic processes implicated in brain injury. MiR-124 regulates glutamate homeostasis by increasing the exposure of EAAT1/2 at the cell membrane. By inhibiting LCN2, miR-138-5p promotes astrocytic survival and limits the release of pro-inflammatory cytokines. Likewise, miR-146 inhibits IRAK1-mediated activation of the NF-κB pathway, attenuating inflammation. MiR-133 suppresses CTGF, a mediator of reactive gliosis, induces the secretion of neurotrophic factors through glial EVs, and limits CSPG-inhibition of neural regeneration. Finally, in mice, miR466m-5p increases the expression of antioxidant genes by inhibiting Keap1 and Bach1, both repressors of the Nrf2 pathway. miR466q and miR467f target MAPK11, therefore reducing the expression of IL-1β and TNFα. Image created with Biorender.com

EVs released by MSCs exert many of their paracrine effects through their miRNA cargoes. These miRNAs regulate key disease mechanisms that link neurodegeneration to chronic neuroinflammation. As extensively discussed, neuronal stress and loss are partially driven by the direct effect of misfolded and aggregated proteins, but mostly by activation of the resident glial cells and, in many settings, by peripheral immune infiltration across the BBB [3, 215]. Accordingly, MSC-EV miRNAs should be considered not only for direct neurotrophic and anti-apoptotic actions, but also for their capacity to reprogram glial and immune pathways that shape the inflammatory microenvironment. The following section highlights specific EV-associated miRNAs that contribute to these therapeutic effects **(**Fig. 4**)**.

Among the miRNAs responsible for direct neuroprotection, anti-apoptotic outcomes, and neurogenesis, miR-21-5p is the most abundant in BM-MSC-derived EVs [216] and targets key pro-apoptotic pathways, including the TRPM7 (transient receptor potential melastatin 7) ion channel which is involved in oxidative stress [217, 218]. Other mechanisms rely on the downregulation of FasL [219] and the modulation of Bcl-2 family proteins, upregulating Bcl-2 protein level while downregulating the Bax (Bcl-2-associated X protein) level [216, 220]. MiR-133 promotes neurogenesis and neurorepair through regulating the dopaminergic lineage specification [221, 222] and attenuating inhibition of the axonal growth caused by the connective tissue growth factor (CTGF) released by astrocytes [223, 224]. It also supports neuronal differentiation and maturation by inhibiting the RAS homolog gene family member A (RhoA), thereby activating the ERK 1/2 pathway and the transcription profile promoted by cyclic adenosine monophosphate (cAMP) response element binding protein (CREB) [225–227]. Dendritic plasticity and neurogenesis are also mediated by miR-17–92 and miR-138. The former is responsible for the silencing of the phosphatase and tensin homolog (PTEN) protein, which, in turn, activates the Akt/GSK3β signalling [228, 229]. The latter represses RhoA by targeting acyl-protein thioesterase 1 (APT1), which antagonizes dendritic spine growth [230, 231]. Finally, miR-29b mitigates neuronal apoptosis [232] and enhances axonal regeneration [233, 234], while miR-124 downregulates SRY-box transcription factor 9 (SOX9) [235], elevating neuronal fate determination and expression of the neuron-associated stemness markers Nestin and SOX2 [236].

Some of the miRNAs that directly enhance neuronal survival can also target inflammatory pathways. MiR-21 can inhibit NF-κB signalling in neurons and astrocytes, hindering IκBα phosphorylation and p65 nuclear translocation [237]. MiR-138 can suppress the secretion of Lipocalin-2 by reactive astrocytes, a potent neurotoxic factor and a link between glial-promoted inflammation and neuron vulnerability [238]. MiR-30 downregulates autophagy-related genes, such as Atg5 (autophagy-related gene 5), microtubule associated protein 1 light chain 3, and Beclin 1 [239–241], while limiting microglial M1 polarization [239]. MiR-146a reduces inflammation, inhibiting interleukin-1 receptor-associated kinase 1 (IRAK1)-mediated NF-κB activation in astrocytes [242, 243]. MiR-124 enhances glutamate uptake, thereby reducing excitotoxicity, and can shift microglial polarization toward the reparative M2 phenotype [244, 245]. Finally, studies involving murine BM-MSCs highlighted interesting roles in glial-driven inflammation. MiR-466 m-5p was shown to target repressors of antioxidant gene expression that interfere with the nuclear factor erythroid 2-related factor 2 (Nrf2) pathway [246]. MiR-466q and miR-467f can inhibit TNF-α and IL-1β expression by repressing mitogen-activated protein kinase 11 (MAPK11) in astrocytes, while in microglia they target upstream regulators of p38 and MAPK signalling [247].

## Therapeutic effects of MSCs in preclinical models of NNDs

### MSCs in PD

Despite early evidence suggesting neuronal integration, the prevailing mechanism by which MSCs enhance motor function in PD remains to be paracrine modulation and immunoregulation. In 2012, Yao and colleagues pre-conditioned NSCs with conditioned medium from BM-MSCs before transplantation into 6-OHDA mice. This approach enhanced the dopaminergic differentiation of NSCs and promoted their integration into host neural circuits, leading to functional recovery and increased survival [248]. Schwerk and colleagues utilized AD-MSCs to promote robust reactive neurogenesis in the subventricular zone and to alleviate Parkinsonian symptoms. These effects were attributed to neurotrophic factors and anti-inflammatory cytokines released by AD-MSCs, which limited neuroinflammation and neuronal death, ultimately improving locomotor function [249].

### MSCs in MS

MSCs have also shown therapeutic effects in EAE. Zappia and colleagues demonstrated that intravenous administration of autologous BM-MSCs markedly improved disease outcomes in this model [250]. This effect was attributed to the prevention of demyelinating degeneration through reduced cell infiltration in the brain and spinal cord, as well as to the in vivo induction of T cell anergy [250]. In addition, BM-MSCs exert systemic immunoregulatory effects while residing in lymphoid tissues, but can also reach sites of CNS inflammation. Once in the CNS, they protect neurons from death and may also acquire a neuron-like phenotype, as evidenced by β3-tubulin expression, thereby contributing to disease amelioration [251]. However, the notion that MSCs can directly differentiate into neurons and replace damaged cells remains controversial [252]. It is more widely accepted that MSCs may stimulate the endogenous neural stem cell pool within the CNS. Accordingly, Bai and colleagues assessed cellular composition in neurospheres generated from subventricular NSCs of control and BM-hMSC-treated EAE mice. In untreated mice, neurospheres were predominantly composed of astrocytes, with minimal differentiation into oligodendrocytes or neurons, indicative of reactive astrogliosis. In contrast, neurospheres from BM-MSC-treated mice displayed enhanced neuronal differentiation and a higher proportion of mature oligodendrocytes, correlated with improved in vivo myelination [253]. Multiple studies further showed that the immunomodulatory effects of MSCs rely on a shift in immune responses, suppressing Th1 differentiation and pro-inflammatory cytokine production [181] while promoting a Th2-skewed response, characterized by anti-inflammatory cytokines such as IL-4 and IL-5 [253]. MSCs strongly suppress Th17 cells, a subset now recognized to be central to MS pathogenesis [254]. Inhibiting HGF or blocking its receptor, mesenchymal-epithelial transition factor (c-Met), almost completely abolished the therapeutic benefits of MSCs in EAE models. HGF is indeed involved in remyelination, oligodendrocyte and neuronal maturation, and the restoration of motor function [255].

In 2018, Laso-García and colleagues demonstrated that EVs derived from AD-MSCs significantly attenuated brain tissue degeneration and curbed the aberrant inflammatory response in a murine model of TMEV (Theiler’s murine encephalomyelitis virus)-induced demyelinating syndrome [256]. The treatment promoted neurogenesis and improved motor function. At the spinal cord level, the EVs mitigated pro-inflammatory microglial activation and, systemically reduced the frequencies of Th1 and Th17 lymphocytes [256]. Clark and colleagues proved that EVs from chorionic villi-derived MSCs reduced axonal damage and neuronal death by promoting oligodendrogenesis and remyelination in EAE mice. Riazifar and colleagues demonstrated that the intravenous administration of IFNγ-primed BM-MSCs not only decreased neuroinflammation and reduced demyelination, but also significantly increased the number of immunoregulatory CD4⁺CD25⁺FoxP3⁺ regulatory T cells in the spinal cords of EAE mice [257]. AD-MSCs engineered for co-expressing LIF (leukemia inhibitory factor) and IFN-β showed similar results, with enhanced remyelination, increased number of Olig2⁺ oligodendrocyte progenitors, and higher MBP expression [258].

### MSCs in AD

Recent studies have also shown therapeutic effects of MSCs in AD. For example, BM-MSC transplantation significantly reduced tau hyperphosphorylation in 3 × Tg-AD mice by reducing inflammation, including downregulation of GFAP expression in astrocytes and Iba-1 (ionized calcium-binding adapter molecule 1) expression in microglia [259]. Similarly, intracerebral injection of BM-MSCs led to a significant reduction in Aβ plaque burden and improved cognitive performance in APP/PS1 mice. These effects were associated with the modulation of defective microglia activation, favoring their phagocytic capacity without the neurotoxic pro-inflammatory reaction [260]. Placenta-derived MSCs (PD-MSCs), isolated from the amniotic-chorionic membrane, exhibited neuroprotective effects in mice infused with Aβ_1–42_, improving cognitive function as assessed by the Morris water maze and passive avoidance tests [261]. This behavioral improvement was associated with downregulation of amyloid progenitor precursor (APP) protein, BACE1 (beta-site amyloid precursor protein cleaving enzyme), and Aβ expression, as well as reduced β- and γ-secretase activity. Moreover, PD-MSC transplantation significantly suppressed glial activation and reduced the expression of pro-inflammatory inducible nitric oxide synthase and cyclooxygenase-2. PD-MSCs prevented neuronal loss and promoted the differentiation of neuronal progenitor cells into mature neurons [261]. UC-MSCs enhanced cognitive function of 5 × FAD mice by promoting hippocampal neurogenesis and attenuating tau hyperphosphorylation through secretion of galectin-3 and growth differentiation factor-15 [262, 263].

**Fig. 5:**
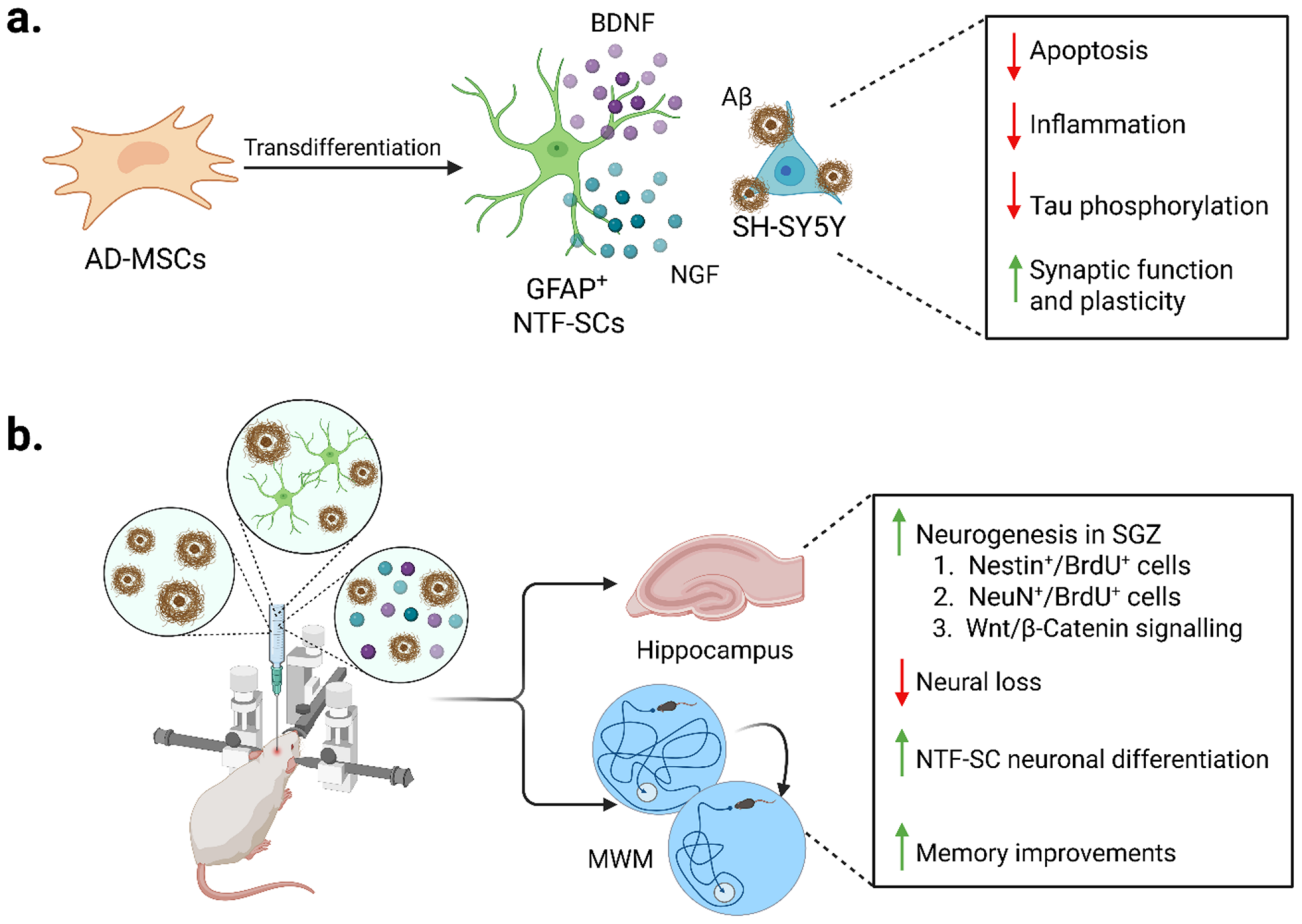
In vitro and in vivo neuroprotective effects of NTF-SCs in a mouse AD model. **a** AD-MSCs can be transdifferentiated into GFAP⁺ NTF-SCs, which act as a reservoir of neurotrophic factors such as BDNF and NGF. In vitro, these cells can mitigate Aβ-induced neurotoxicity in SH-SY5Y neurons, reducing apoptosis, inflammation, and tau phosphorylation while enhancing synaptic function and plasticity. **b** In vivo transplantation of NTF-SCs into the hippocampus of AD mice reduces Aβ burden and neural loss, promotes neurogenesis in subgranular zone (SGZ), and increases the numbers of Nestin⁺ and NeuN⁺ neurons, while enhancing Wnt/β-catenin signaling. NTF-SCs also show partial neuronal differentiation and contribute to cognitive improvements

Other studies supported the therapeutic effects of MSC EVs. Intranasally administered BM-MSC-derived EVs improved temporal and spatial cognitive function and reduced astrocytic activation [264]. EVs from BM-MSCs engineered to increase brain tropism in order to facilitate intravenous administration, showed similar effects, accompanied by decreased plaque deposition [265]. A study by Jahed and colleagues demonstrated how MSC plasticity may pave the way for advanced cell therapies. While MSCs inherently possess immunomodulatory properties, the levels of their physiological secretion of specific neurotrophins may not be sufficient to halt severe, progressive neurodegeneration or promote robust synaptic repair. To address this limitation, the researchers induced transdifferentiation of AD-MSCs into “neurotrophic-secreting stem cells” (NTF-SCs), which are biologically engineered to secrete higher quantities of neurotrophic factors. These NTF-SCs displayed an astrocyte-like morphology and could secrete neurotrophic factors, such as BDNF and nerve growth factor (NGF). In experimental models, NTF-SCs were co-cultured with SH-SY5Y cells pre-treated with recombinant human Aβ_1–42_ to mimic AD-like conditions. Comparative analyses confirmed that NTF-SCs were more effective than undifferentiated MSCs in increasing NGF and BDNF release and attenuating Aβ-induced toxicity. The results also showed a significant enhancement in neuronal survival, accompanied by a general reduction in neuronal inflammatory activation. Moreover, a decrease in tau phosphorylation was documented, alongside the upregulation of genes of proteins associated with synaptic and cellular plasticity in SH-SY5Y cells, such as synapsyn-1, synaptophysin, and NGFI-A (nerve growth factor-induced gene A) [266] **(**Fig. 5a**)**. Subsequently, Bahlakeh and colleagues assessed the therapeutic potential of NTF-SCs in Balb/c mice injected with Aβ_1–42._ They demonstrated that the transplanted cells promoted endogenous neurogenesis, resulting in a marked improvement of memory performance [267] **(**Fig. 5b**)**.

## Clinical applications of MSCs in NNDs

Building on the extensive preclinical evidence elucidating the mechanisms by which MSCs promote neuronal survival in neurodegenerative and neuroinflammatory contexts, several phase I/II clinical trials have been conducted to assess their safety in patients and to begin exploring potential efficacy on motor, cognitive, and biochemical outcomes **(**Table 1**)**. For PD, to date, there is only one completed phase I trial with published results [155]. Autologous BM-MSCs were administered intravenously at different doses, demonstrating an excellent safety and immunogenicity profile, even at the highest dose [155]. The highest tested dose appeared to reduce peripheral inflammation, as evidenced by the downregulation of several pro-inflammatory cytokines and consequent motor improvements. At the same time, the serum level of BDNF was increased, suggesting neurotrophic support. Based on these findings, a subsequent phase II trial involving a larger cohort of patients was designed to evaluate the impact of MSC administration on quality-of-life measures (NCT04506073). However, results from this study, as well as from another phase II trial using autologous AD-MSCs (NCT04928287), have not yet been published. In AD, results from phase I, II, and follow-up studies employing allogeneic UC-MSCs (NEUROSTEM®-AD) delivered intrathecally are already available [156, 268]. Despite the more invasive administration route, the treatment was well tolerated, without dose-limiting toxicities, and showed promising efficacy in patients with mild to moderate cognitive and psychiatric impairment. Notably, AD-related biomarkers, including Aβ_1–42_, total and hyperphosphorylated tau protein, transiently reduced in CSF samples, as well as leucocytosis, further support the involvement of immunomodulatory and paracrine pathways as contributing mechanisms of action. Autologous BM-MSCs (Lomecel-B) [269] and AD-MSCs (Astro-Stem; NCT03117738) have also been tested via intravenous infusion. Encouraging safety outcomes have been reported for BM-MSCs, along with preliminary cognitive benefits attributed to their pro-angiogenic and immunomodulatory properties. Currently, the largest number of clinical results are available from phase I and II trials in MS. Most of the trials employed autologous BM-MSCs [157, 158, 270–278], in some cases engineered to act as reservoirs of neurotrophic factors [279] or committed toward a neural progenitor-like phenotype [280, 281]. Across the different trials, a consistent finding is the favourable safety and tolerability profile of MSC administration, regardless of the delivery route, with adverse drug reactions generally mild and never severe enough to compromise clinical feasibility [270, 271, 274–277]. Several studies also reported motor improvements [270–272, 279–281]. These were accompanied by changes in CSF composition, including a decrease in neurofilament levels as a proxy of neuronal damage [271, 272, 279, 281]. Moreover, paracrine modulation of peripheral immunity was observed, characterized by a reduction in Th1 lymphocytes [274] and a transient increase in regulatory T cells [270, 273]. Other investigations described benefits at the level of demyelinating plaques, with stabilization of existing lesions and absence of new lesion formation, suggesting a lack of disease reactivation [157, 271, 273, 280]. Conversely, the MEsenchymal StEm cells for Multiple Sclerosis (MESEMS) trial, a large multicentre study, confirmed the excellent safety profile of BM-MSCs but failed to meet secondary efficacy endpoints, as no significant improvements in disease progression or motor function were observed [158, 278]. Additional sources of MSCs, including adipose tissue [282] (NCT05116540) and perinatal tissues [283–286], have also been tested, consistently achieving primary safety outcomes and, in some cases, providing functional benefits such as improved motor performance, better bladder function control, and stabilization of cerebral lesion activity.

**Table 1:** Clinical trials testing MSC-based therapies for treating the three most prevalent neurodegenerative diseases worldwide

Disease	Study ID & title	Study design	Treatment	Dose (cells)	Route & administration schedule	Number of patients	Location	Dates	Outcomes	Results	Reference
Parkinson’s disease (PD)	NCT02611167 *Pilot phase I study of allogeneic bone marrow-derived mesenchymal stem cell therapy for idio-pathic Parkinson’s disease*	Phase 1; open-label, dose-escalation study	Allogenic BM-MSCs	1 × 10^6^/kg 3 × 10^6^/kg 6 × 10^6^/kg 1 × 10^7^/kg	Intravenous; 1 dose	20	The University of Texas Health Science Center at Houston. Houston, Texas, US	Nov 2017-Sep 2019	To assess safety, feasibility, and efficacy of infusions. To evaluate the treatment impact on motor and behavioral features, life quality, brain activity and soluble markers	A single dose is safe, well tolerated and not immunogenic. The highest dose efficiently reduced peripheral inflammatory markers and improved motor function due to immunomodulation and neurotrophic support	[155]
	NCT04506073 *A randomized, double-blind, placebo-controlled trial of allogeneic bone marrow-derived mesenchymal stem cells as a disease-modifying therapy for idiopathic Parkinson’s disease*	Phase 2; randomized, double-blind, placebo-controlled study	Allogenic BM-MSCs	1 × 10^7^/kg	Intravenous; twice and 1 placebo infusion or trice every 4 months	45	The University of Texas Health Science Center at Houston. Houston, Texas, US	Nov 2020-Jul 2023	To test safety and tolerability in terms of adverse drug reactions (ADRs) and immunogenicity. To evaluate its impact on motor and cognitive functions, life quality and CSF/blood biomarkers	No results published yet	–
	NCT04928287 *A randomized, double-blind, single center, phase 2, efficacy and safety study of autologous HB-adMSCs versus placebo for the treatment of patients with Parkinson’s disease*	Phase 2; randomized, double-blind, placebo-controlled study	HB-adMSCs (Autologous AD-MSCs)	2 × 10^8^	Intravenous; 6 doses at week 0, 4, 8, 16, 24 and 36	24	Hope Biosciences Stem Cell Research Foundation. Sugar Land, Texas, US	Jun 2021-Feb 2023	To test the administration impact on metabolic and blood parameters of patients. To assess its physical, mental, and social effects	No results published yet	–
Alzheimer’s disease (AD)	NCT01297218 *Open-label, single-center, phase 1 clinical trial to evaluate the safety and the efficacy of NEUROTSTEM®-AD in patients with dementia of the Alzheimer's type*	Phase 1; open-label study	NEUROSTEM®-AD (UC-MSCs)	Low dose: 3 × 10^6^ High dose: 6 × 10^6^	Intrathecal; 1 dose	9	Samsung Medical Center. Seoul, Korea, Republic of	Feb 2011-Dec 2011	To assess safety and the maximum tolerated dose (MTD). To evaluate its efficacy in improving cognitive functions in AD patients	Hippocampal and precuneus stereotactic administrations of UC-MSCs are feasible, safe, and well tolerated in patients with mild-to-moderate AD dementia	[268]
	NCT02600130 *A phase, I prospective, randomized, double-blinded, placebo-controlled, trial to evaluate the safety and potential efficacy of lomecel-B infusion versus placebo in patients with Alzheimer's disease*	Phase 1; prospective, randomized, placebo-controlled, double-blinded study	Lomecel-B (Autologous BM-MSCs)	Low dose: 2 × 10^7^ High dose: 1 × 10^8^	Intravenous; 1 dose	33	Brain Matters Research. Delray Beach, Florida, US; University of Miami Miller School of Medicine. Miami, Florida, US; Miami Jewish Health. Miami, Florida, US	Oct 2016-Sep 2021	To evaluate safety and primary efficacy in improving cognitive functions, quality life and blood/CSF markers in AD patients	Good tolerability and preliminary positive effects on biomarkers and cognitive function, supporting its potential pro-vascular and anti-inflammatory mechanisms	[269]
	NCT02054208 *A double-blind, single-center, phase 1/2a clinical trial to evaluate the safety and exploratory efficacy of intraventricular administrations of NEUROSTEM® versus placebo via* * an Ommaya reservoir in patients with Alzheimer's disease*	Phase 1/2; randomized, placebo-controlled, double-blinded study	NEUROSTEM®-AD (UC-MSCs)	Low dose: 1 × 10^7^ High dose: 3 × 10^7^	Intracerebroventricular; every four weeks for 3 doses	9	Samsung Medical Center. Seoul, Korea, Republic of	Mar 2014-Dec 2019	To evaluate dose limiting toxicity (DLT) and treatment efficacy in improving cognitive functions, CSF markers and MRI outcomes in AD patients	Moderate and transient ADR (fever, headache, nausea, and vomiting), but no dose-limiting toxicity. Temporary CSF markers improvement and CSF leukocytosis	[156]
	NCT03172117 *Follow-up study of safety and efficacy in subjects who completed NEUROSTEM® phase-I/IIa clinical trial*	Long-term follow-up study for up to 36 months	NEUROSTEM®-AD (UC-MSCs)	Low dose: 1 × 10^7^ High dose: 3 × 10^7^	Intracerebroventricular; every four weeks for 3 doses	9	Samsung Medical Center. Seoul, Korea, Republic of	May 2019-Mar 2022	To evaluate DLT and treatment efficacy in improving cognitive functions, CSF markers and MRI outcomes in AD patients	Moderate and transient ADRs (fever, headache, nausea, and vomiting), but no dose-limiting toxicity. Temporary CSF markers improvement and CSF leukocytosis	[157]
	NCT04040348 *A phase I, prospective, open-label trial to evaluate the safety, tolerability and exploratory outcomes of multiple allogeneic human mesenchymal stem cells (HMSC) infusions in patients with mild to moderate Alzheimer’s disease*	Phase 1; prospective, open-label study	UC-MSCs	1 × 10^7^	Intrathecal	6	University of Miami. Miami, Florida, US	Oct 2019-Apr 2023	To assess safety, side effects and its effectiveness in terms of neuropsychiatric and cognitive functions, CSF/blood biomarkers and hippocampal volume	No results published yet	–
	NCT03117738 *A phase 1/2, randomized, double-blind, placebo-controlled study to evaluate the safety and efficacy of astrostem, autologous adipose tissue derived mesenchymal stem cells, in patients with Alzheimer’s disease*	Phase 1/2; randomized, double-blind, placebo-controlled, parallel-group comparison study	AstroStem (Autologous AD-MSCs)	2 × 10^8^ AD-MSCs	Intravenous; every two weeks for 10 doses	21	ATP Clinical Research. Costa Mesa, California, US Syrentis Clinical Research. Santa Ana, California, US Valden Medical. Honolulu, Hawaii, US	May 2017-Aug 2019	To test safety and neuropsychiatric and cognitive improvements	No results published yet	–
Multiple sclerosis (MS)	NCT00813969 *A phase I study to assess the feasibility, safety, and tolerability of autologous mesenchymal stem cell transplantation in patients with relapsing forms of multiple sclerosis*	Phase 1; open label, prospective study	Autologous BM-MSCs	2 × 10^6^/kg	Intravenous; 1 dose	24	Cleveland Clinic Mellen Center. Cleveland, Ohio, US	Mar 2011-May 2014	To evaluate the feasibility of culturing BM-MSCs, and infusion-related safety and tolerability. To evaluate effects on MRI-quantified lesions	Well tolerated without treatment-related severe or serious adverse events, or evidence of disease activation	[157]
	NCT03778333 *Mesenchymal stem cells for progressive multiple sclerosis_Sweden*	Phase 1; open label, prospective study	Autologous BM-MSCs	1–2 × 10^6^/kg	Intravenous, 1 dose	7	Karolinska Institute, Karolinska University Hospital. Stockholm, Sweden	Dec 2012-Dec 2016	To define treatment feasability, and impact on disability and peripheral immune response	Well tolerated during clinical remission. MRI-tested absence of new lesions and no disability worsening. Post-infusion increasing in proportion of FOXP3 + Tregs	[273]
	NCT06360861 *An open-label, non-randomized, phase I study of allogeneic placenta derived mesenchymal stem cells in patients with secondary-progressive multiple sclerosis (SPMS)*	Phase 1; open label, prospective study	PD-MSCs	3 × 10^6^/kg	Intravenous, 1 dose	5	Tehran University of Medical Sciences,Tehran, Iran. Tehran, Iran, Islamic Republic of	Jul 2019-Mar 2024	To define safety and effects on cognitive, brain and motor performances. Assessment of its roles in immunomodulation, visuospatial and verbal activity	No significant ADRs. Sustained improvements in clinical, cognitive and psychological outcomes. Enhancements in brain connectivity, decrease in CD20/CD19 B cell markers and increase in IL-10, alongside reduction in pro-inflammatory cytokines (IL-6, TNFα and IL-17)	[284]
	NCT03117738 *Phase 1 safety study of autologous bone marrow-derived mesenchymal stem cell-derived neural progenitor cells (MSC-NP), expanded* ex vivo, *administered intrathecally in pa-tients with multiple sclerosis*	Phase 1; open label, prospective study	Autologous MSC-NPs (Mesenchymal stem cell-neural progenitors)	Low dose: 2 × 10^6^ High dose: 1 × 10^7^	Intravenous; 3 doses in 3 months	20	Tisch MS Research Center of New York. New York, New York, US	Apr 2014-Mar 2013	To define ADR rate and to measure brain activity, quality of life and disability scores	No serious adverse effects. Improvements in muscle strenght, bladder function, deambulation and disability severity	–
	NCT02239393 *Mesenchymal stem cell therapy for Canadian MS patients*	Phase 1/2; randomized, double-blinded, placebo-controlled, cross-over study	Autologous BM-MSCs	1–2 × 10^6^/kg	Intravenous; 2 doses with crossing the groups over after 24 weeks with either MSCs or placebo	31	Health Sciences Centre. Winnipeg, Manitoba, Canada; Ottawa Hospital—General Campus. Ottawa, Ontario, Canada	Jun 2015-Dec 2019	To evaluate treatment safety and MRI-tested efficacy. To assess administration efficacy in limiting replases and disability progression	Discreet tolerability, but no-significant reduction in brain lesions and no clinical differencies between groups*	[158, 278]
	NCT04823000 *Long term clinical and immunological effects of repeated mesenchymal stem cells (MSC) injections in patients with progressive forms of multiple sclerosis (MS)*	Phase 1/2; open label, prospective study	Autologous BM-MSCs	1 × 10^6^/kg	Intrathecal or intravenous; 9 doses in total every 6–12 months	24	Hadassah Medical Organization. Jerusalem, Israel	Jan 2013-Apr 2020	To evaluate motor stability of patients after treatment, its safety and peripheral immunlogical outcomes	No treatment-related ADRs. Motor improvement in half of the participants. Transient upregulation of CD4^+^ CD25^+^ FOXP3^+^ T cells and reduced lymphocyte proliferation capacity	[270]
	NCT01606215 *Stem cells in rapidly evolving active multiple sclerosis (STREAMS)*	Phase 1/2; randomized, double-blinded, placebo-controlled, study	Autologous BM-MSCs	1–2 × 10^6^/kg	Intravenous; 1 dose	21	Imperial College Healthcare NHS Trust. London, United Kingdom	Jan 2013-Aug 2019	To value treatment safety and new brain lesions, relapses and the disability progression rates	Discreet tolerability, but no-significant reduction in brain lesions and no clinical differencies between groups*	[158, 278]
	NCT01730547 *Phase 1/2 clinical trial with autologous mesenchymal stem cells for the therapy of multiple sclerosis*	Phase 1/2; randomized, double-blinded, placebo-controlled, cross-over study	Autologous BM-MSCs	1–2 × 10^6^/kg	Intravenous; 1 dose	2	Karolinska Institute, Karolinska University Hospital Solna. Stockholm, Sweden	Feb 2013-Nov 2021	To determine safety and to obtain preliminary data on efficacy in terms of combined MRI activity and clinical efficacy	Discreet tolerability, but no-significant reduction in brain lesions and no clinical differencies between groups*	[158, 278]
	NCT01895439 *Phase II study: use of autologus mesenchymal stem cells in multiple sclerosis patients who do not respond to conventional treatment*	Phase 1/2; open label, prospective study	Autologous BM-MSCs	1.1 × 10^8^	Intrathecal, 1 dose	10	Cell Therapy Center, Jordan University Hospital. Amman, Jordan	Oct 2012-Feb 2016	To assess treatment safety and MRI- or ophtalmologically-tested therapeutic benefits	Safe and well tolerated. Functional and clinical amelioration, not related to worsening in lesion load	[272]
	NCT02034188 *Feasibility study of human umbilical cord tissue-derived mesenchymal stem cells in patients with multiple sclerosis*	Phase 1/2; open label, prospective study	UC-MSCs	2 × 10^7^	Intravenous, 7 doses over a week, once per day	20	Stem Cell Institute. Panama City, Panama	Jan 2014-Mar 2013	To test freedom from treatment-associated ADR, administration efficacy in improving quality of life and disease-related disability	Improvements in all tested clinical outcomes and quality of life. Positive patient perspective of a significant health change	[283]
	NCT00395200 *Autologous adult human mesenchymal stem cells: a neuroprotective therapy for multiple sclerosis*	Phase 1/2; open label, prospective study	Autologous BM-MSCs	6 × 10^6^/kg	Intravenous, 1 dose	10	University College London Institute of Neurology. London, UK; University of Cambridge Dept of Clinical Neurosciences. Cambridge, Cambridgeshire, UK	Jul 2008-Dec 2013	To define safety and feasability, and impact on visual, motor and MRI-assessed brain activity	No serious ADRs, progress in visual acuity and visual evoked response latency, with increased optic nerve area. Reduced general disability progression	[275, 276]
	NCT01377870 *Effect and side effect of mesenchymal stem cell in multiple sclerosis*	Phase 1/2; randomized, double-blinded, placebo-controlled study	Autologous BM-MSCs	Not specified	Intravenous; 2 doses with crossing the groups over after 24 weeks with either MSCs or placebo	22	Royan Institute. Tehran, Iran, Islamic Republic of	Dec 2011-Apr 2014	To test treatment impact on brain, disease relapses and patient disability	No results published yet	–
	NCT00781872 *Explorative trial to investigate the safety and clinical effects of autologous mesenchymal bone marrow stem cells (MSC) following their intrathecal and intravenous administration in severe cases of multiple sclerosis (MS)*	Phase 1/2; open label, prospective study	Autologous BM-MSCs	Intrathecally: 6 × 10^7^ Intravenously: 2 × 10^7^	Intrathecal or intravenous; 1 dose	15	Hadassah-Hebrew University Hospital. Jerusalem, Israel	Oct 2006-Dec 2009	To test treatment impact on brain, disease relapses and patient disability	No severe treatment-related ADRs. Increased CD4^+^ CD25^+^ Tregs and decreased proliferative responses of lymphocytes. Less pronounced expression of costimulatory and HLA-DR molecules on DCs	[277]
	NCT01745783 *Clinical trial phase I/II multicenter, randomized, crossover, double-blind evaluation of the safety and feasibility of systemic therapy with mesenchymal cells derived from autologous bone marrow in patients with multiple sclerosis*	Phase 1/2; randomized, double-blinded, placebo-controlled, crossed over study	Autologous BM-MSCs	1–2 × 10^6^/kg	Intravenous; 2 doses with crossing the groups over after 3 months with either MSCs or placebo	24	University Hospital Reina Sofia. Córdoba, Spain; University Regional Hospital Carlos Haya. Málaga, Spain; University Hospital Virgen Macarena. Sevilla, Spain	Sep 2017-Jan 2020	To assess unexpected and serious ADR. And to determine if there are differences between placebo and treatment in terms of disease activity	Discreet tolerability, but no-significant reduction in brain lesions and no clinical differencies between groups*	[158, 278]
	NCT03326505 *The effect of stem cell therapy and comprehensive physical therapy in motor and non-motor symptoms in patients with multiple sclerosis: a comparative study*	Phase 1/2; randomized, single-blinded study	UC-MSCs	Dose I: 1 × 10^8^ intrathecally and 5 × 10^6^ intravenously; dose II: the same as dose I after 1 month; dose III: 8–10 mL of UC-MSC-derived CM intrathecally and 3 months later	Intrathecal and intravenous; 3 doses for group A or 2 doses for group B (without dose II)	60	Cell Therapy Center, University of Jordan. Amman, Jordan	Jan 2013-Jun 2020	To test severe ADR, clinical and biological outcomes	Both treatments are safe. Improvements in general disability for all patients. Better outcomes for group A regarding lesion load, cortical thickness, manual dexterity, and information processing speed. Inflammation-related and antigen-presenting genes were downregulated in both groups. Some genes, such as TNFα, TAP-1, and miR142 were downregulated only in group A	[285, 286]
	*Phase I-II clinical trial with autologous bone marrow derived mesenchymal stem cells for the therapy of multiple sclerosis*	Phase 1/2; randomized, double-blinded, placebo-controlled, cross-over study	Autologous BM-MSCs	1 × 10^6^/kg	Intravenous; 2 doses with crossing the groups over after 24 weeks with either MSCs or placebo	9	Germans Trias i Pujol Hospital. Badalona, Barcelona, Spain	Dec 2013-Jul 2016	To evaluate treatment safety and MRI-tested efficacy. To assess changes in disability, in quality of life, the immunological outcomes and repair effects on axons	Discreet tolerability, but no-significant reduction in brain lesions and no clinical differencies between groups*	[158, 278]
	NCT01056471 *Multicenter clinical trial phase I/II randomized, placebo-controlled study to evaluate safety and feasibility of therapy with two different doses of autologous mesenchymal stem cells in patients with secondary progressive multiple sclerosis who do not respond to treatment*	Phase 1/2; randomized, triple-blinded, placebo-controlled study	Autologous AD-MSCs	Low dose: 1 × 10^7^/Kg High dose: 4 × 10^7^/kg	Intravenous; 1 dose	30	Hospital Regional Universitario de Málaga. Málaga, Spain Hospital Universitario Virgen Macarena Sevilla, Spain	Jan 2010-Jun 2016	To test safety. To analyze its impact on the quality of life and clinical, imaging, immunological and neurophysiological changes	No severe treatment-related complications. No statistically significant changes in treatment biomarkers, in number of MRI-tested brain lesions and in disability scores	[282]
	NCT02495766 *Treatment of autologous mesenchymal stem cells derived from bone marrow as a potential therapeutic strategy for the treatment of multiple sclerosis*	Phase 1/2; randomized, double-blinded, placebo-controlled, crossed-over study	Autologous BM-MSCs	Not specified	Not specified; 2 doses with crossing the groups over after 6 months for a re-treatment with either MSCs or placebo	8	Hospital Vall Hebron. Barcelona, Spain	May 2015-Nov 2018	To test safety profile, disability scores, MS outbreaks and MRI-visualized number of brain lesions	No results published yet	–
	NCT01228266 *Autologous mesenchymal stem cell transplantation in multiple sclerosis: a randomized, double-blind, crossover with placebo phase II study*	Phase 2; randomized, double-blinded, placebo controlled, crossed-over study	Autologous BM-MSCs	2 × 10^6^/kg	Intravenous; 2 doses with crossing the groups over after 6 months with either MSCs or placebo	9	Neurology Service, Hospital Clinic de barcelona. Barcelona, Spain	Dec 2010-Dec 2013	To evaluate safety and MRI-tested efficacy. To test it impact on the qualty of life peripheral immunlogical outcomes	No ADRs, reduced new forming brain lesions and a slight, statistically non-significant decrease of Th1 cells in blood	[274]
	*A randomized, double-blind, single-center, phase 2, efficacy and safety study of autologous HB-adMSCs versus placebo for the treatment of patients with multiple sclerosis*	Phase 2; randomized, double-blinded, placebo controlled study	HB-adMSCs (Autologous AD-MSCs)	Dose I: 1 × 10^8^ intrathecally and 5 × 10^6^ intravenously; dose II: the same as I dose after 1 month; dose III: 8–10 mL of UC-MSC-derived CM intrathecally	Intravenous; 6 doses over 52 weeks	24	Hope Biosciences Stem Cell Research Foundation. Sugar Land, Texas, US	Nov 2021-Jun 2024	To assess treatment tolerability. To evaluate its impact on motor, cognitive and behavioural aspects, as well as cardiac function and blood/metabolism markers	No results published yet	–
	NCT03799718 *A phase 2 open-label multicenter study to evaluate the safety and efficacy of repeated administration of NurOwn® [autologous mesenchymal stem cells secreting neurotrophic factors (NTF), MSC-NTF] cells in participants with progressive MS*	Phase 2; open label, prospective study	NurOwn® (Autologous BM-MSCs secreting neurotrophic factors (NTF), MSC-NTF	1–1.25 × 10^8^	Intrathecal; 3 doses over 16 weeks	18	University of Southern California. Los Angeles, California, US; Stanford University School of Medicine. Redwood City, California, US; The Mount Sinai Hospital. New York, New York, US; Cleveland Clinic. Cleveland, Ohio, US	Mar 2019-Mar 2021	To test treatment-related ADRs. To value motor improvements and changes in CSF neuroprotective and angiogenic biomarkers	No ADRs and disease-related condition worsening. 90% of patients improved their motor acitivity. Increases in CSF neuroprotective factors, and decreases in inflammatory biomarkers	[279]
	NCT03355365 *Autologous, bone marrow-derived mesenchymal stem cell-derived neural progenitor cells (MSC-NP), expanded *ex vivo*; administered intrathecally*	Phase 2; randomized, double-blinded, placebo-controlled, compassionate crossed-over study	Autologous MSC-NPs (Mesenchymal stem cell-neural progenitors)	1 × 10^7^	Intrathecal; 6 doses every two months	54	Tisch MS Research Center of New York. New York, New York, US	Sep 2018-Jun 2023	To test safety. To analyze its impact on the quality of life and clinical, imaging, immunological and neurophysiological changes	No severe ADRs. Improved walking ability of patients requiring aids. Primary motor outcomes were not met, but secondary walking outcomes significantly improved. Further improvememnts in bladder function and MRI-visualized cortical gray matter atrophy. Increased production of MMP9 and decreaded production of CCL2	[280, 281]
	NCT02166021 *Phase 2 trial to investigate the clinical efficacy & the optimal administration (Based on the immunological, clinical & neuroradiological effects) of autologous mesenchymal bone marrow stem cells in active & progressive multiple sclerosis*	Phase 2; randomized, double-blinded, placebo-controlled, cross-over study	Autologous BM-MSCs	1 × 10^6^/kg	Intrathecal or intravenous; 2 doses with crossing the groups over after 6 months for a re-treatment with either MSCs or placebo	48	Hadassah Medical Organization. Jerusalem, Israel	Jan 2015-Dec 2018	To assess safety and neurological efficacy concerning motor and cognitive scores, relapsing rate, immunological outcomes and brain volume and activity	No treatment-related serious ADRs. Significant improvement in ambulation, cognitive functions, brain lesions and soluble biomarkers. Improved outcomes registered for intrathecal administration	[271]

*Research criteria* Parkinson’s disease, Parkinson, Parkinson disease, Alzheimer’s disease, Alzheimer, Alzheimer’s dementia, Multiple sclerosis, Mesenchymal stem cells, Mesenchymal stem cell transplantation, Mesenchymal stem cell therapy, Mesenchymal stromal cells. ClinicalTrials.gov. Date of access: 17/05/2025

PD, Parkinson’s disease; AD Alzheimer’s disease; MS, multiple sclerosis; MSCs, mesenchymal stem/stromal cells; BM-MSCs, bone marrow-derived MSCs; AD-MSCs, adipose tissue-derived MSCs; UC-MSCs, umbilical cord-derived MSCs; PD-MSCs, placental-derived MSCs; NTF, neurotrophic factors; MSC-NPs, mesenchymal stem cell-neural progenitors; ADRs, adverse drug reactions; CSF, cerebrospinal fluid; MTD, maximum tolerated dose; DLT, dose limiting toxicity; MRI, Magnetic resonance imaging

^*^Studies included in the MEsenchymal StEm cells for Multiple Sclerosis (MESEMS) clinical trial

Unfortunately, no phase III clinical trials are registered for MSC-based treatment of NNDs due to a translational bottleneck stemming from critical barriers. Lack of consensus on cell sourcing, processing, and standardization in cell manufacturing remains the major obstacle. Other limitations include profound variability in trial design and endpoints, often unreasonably hard to reach, as well as unresolved regulatory complexities. Uncertainties about the exact mechanisms of action, and the difficulty of finding large cohorts of patients who can be followed for extended periods, complicate the scenario. Eventually, in 2024, the FDA approved Ryoncil (remestemcel-L-rknd), an allogeneic, BM-MSC therapy for steroid-refractory acute graft-versus-host disease, marking a major clinical milestone for cell-based immunomodulatory therapies [287]. To grant future clinical applications in neuropathology, it is necessary to improve the methodologies for MSC manufacturing, employ larger patient cohorts to enhance statistical robustness, refine diagnostic strategies, and deepen our knowledge of mechanisms through which MSCs produce benefits in NDDs patients.

## Conclusions

Despite decades of research, effective disease-modifying treatments for most NNDs remain elusive. This therapeutic void has prompted growing interest in innovative cell-based approaches, with MSCs emerging as a particularly promising platform. The multifaceted biological profile of MSCs, including their anti-apoptotic, immunomodulatory, neuroprotective, and pro-angiogenic properties, enables them to target multiple pathogenic mechanisms simultaneously. Importantly, their low immunogenicity enables allogeneic administration without long-term immunosuppression, a feature that significantly enhances their translational potential. Importantly, MSCs exert their therapeutic effects primarily through the release of soluble factors and EVs, rather than through cellular integration or transdifferentiation. These observations have contributed to a shift toward acellular MSC-derived products, such as secretomes and EVs, which offer advantages in terms of scalability, reduced regulatory burden, and improved reproducibility across manufacturing batches. Additionally, the context-sensitive plasticity of MSCs allows them to modulate their phenotype in response to local environmental cues, potentially enhancing therapeutic precision.

Several phase I and II clinical trials, especially in the context of MS, have consistently demonstrated favourable safety profiles, a finding rarely observed with such reproducibility. Unfortunately, no phase III clinical trials are currently registered for MSC-based treatment of NNDs. This translational bottleneck likely reflects several critical barriers: lack of consensus on cell sourcing and processing, variability in trial design and endpoints, and unresolved manufacturing and regulatory challenges. Addressing these limitations will require scaling clinical efforts through harmonized methodologies and larger patient cohorts to improve mechanistic understanding, statistical robustness, and pathway-specific targeting. Another major limitation is the lack of long-term safety data. While some concerns have been raised regarding the tumorigenic potential of MSCs, these findings are frequently contradicted by an equally substantial body of evidence supporting their safety. Overall, the majority of NNDs are either late-onset or rapidly progressive and fatal in younger individuals. In such contexts, the risk of delayed-onset adverse events may be clinically less relevant than the potential benefits. Eventually, the clinical feasibility of MSCs was concretely attested when, in 2024, the FDA approved Ryoncil (remestemcel-L), an allogeneic BM-MSC therapy for steroid-refractory acute graft-versus-host disease, marking a major clinical milestone for cell-based immunomodulatory therapies.

## Abbreviations

NNDs: Neurodegenerative diseases
CNS: Central nervous system
MSCs: Mesenchymal stromal cells
EVs: Extracellular vesicles
ROS: Reactive oxygen species
IFN-γ: Interferon gamma
IL: Interleukin
TNF-α: Tumor necrosis factor alpha
MMPs: Metalloproteinases
TGF-β: Transforming growth factor beta
AD: Alzheimer’s disease
Aβ: Amyloid-beta
PD: Parkinson’s disease
BBB: Blood brain barrier
C3: Complement component 3
GFAP: Glial fibrillary acidic protein
BDNF: Brain-derived neurotrophic factor
GDNF: Glial cell line-derived neurotrophic factor
STAT3: Signal transducer and activator of transcription 3
CSPGs: Chondroitin sulfate proteoglycans
CCL2: C–C motif chemokine ligand 2
DAAs: Disease associated astrocytes
BMECs: Brain microvascular endothelial cells
FGF: Fibroblast growth factor
Ang-1: Angiopoietin-1
VEGF: Vascular endothelial growth factor
ZO-1: Zonula occludens 1
PKC: Phosphokinase C
MAPK: Mitogen-activated protein kinase
PI3K: Phosphatidylinositol 3-kinase
EAE: Experimental autoimmune encephalomyelitis
MBP: Myelin basic protein
CSF: Cerebrospinal fluid
TCR: T cell receptor
APCs: Antigen presenting cells
CTLA-4: Cytotoxic T-lymphocytes antigen 4
FOXP3: Forkhead box P3
ADCC: Antibody-dependent cell-mediated cytotoxicity
CCR2: C–C motif chemokine receptor 2
NKG2D: Natural killer group 2D
BM-MSCs: Bone Marrow-derived mesenchymal stromal cells
AD-MSCs: Adipose tissue-derived mesenchymal stromal cells
IDO: Indoleamine 2,3-dioxygenase
PGE_2_: Prostaglandin E_2_
HGF: Hepatocyte growth factor
IL-1RA: Interleukin-1 receptor antagonist
UC-MSCs: Umbilical cord-derived MSCs
Akt: Protein kinase B
Bcl-2: B-cell leukemia/lymphoma 2 protein
PTEN: Phosphatase and tensin homolog
CTGF: Connective tissue growth factor
RhoA: Ras homolog gene family member A
CREB: CAMP response element-binding protein
APT1: Acyl-protein thioesterase 1
SOX9: SRY-box transcription factor 9
IRAK1: Interleukin-1 receptor-associated kinase 1
Ub: Ubiquitin
Nrf2: Nuclear factor erythroid 2-related factor 2
MAPK11: MAP kinase 11
PD-MSCs: Placenta-derived MSCs
APP: Amyloid precursor protein
NTF-SCs: Neurotrophic secreting factors
NGF: Nerve growth factor
